# Smartphone Technology for Clinical Communication in the COVID-19 Era: A Commentary on the Concerning Trends in Data Compliance

**DOI:** 10.3389/fdgth.2022.816604

**Published:** 2022-03-22

**Authors:** Bernadette John, Christine McCreary, Anthony Roberts

**Affiliations:** Cork University Dental School and Hospital, University College Cork, Cork, Ireland

**Keywords:** smartphone technology, clinical communication, information governance, data compliance, COVID-19, WhatsApp, education

## Introduction

The COVID-19 global pandemic has rightly been the focus of attention for the healthcare workforce and has accelerated the global proliferation and adoption of smartphone and digital technologies for clinical communication and remote consultation (1, 2). This is an understandable development as functionality that enables dialogue between clinicians *via* Instant Messaging (IM) Applications or Apps (e.g., such as WhatsApp and iMessage), and between clinicians and patients through videoconferencing Apps (e.g., such as Zoom and Skype), has never been more important. Clinicians have drawn on the familiar and simple smartphone technology to overcome the communication barriers required for social distancing, reducing the risks associated with face-to-face contact and optimizing efficiencies in healthcare (1), often without adequate training as to potential clinical safety risks or data security incidents. These interventions have often had efficiency of communication at their core but may leave legacy issues that will become apparent as the health emergency passes. These channels have permanently and positively transformed communications, although they also raise professional, ethical, security and legal concerns that are associated with these technologies (3, 4). Clinicians (and their patients) may be in danger of becoming “*ensnared”* in a web of hidden data harvesting. Busy clinicians using smartphones to communicate effectively within their own healthcare teams or directly with patients, could face negative ramifications of these technologies unless the Information Commissioners Office (ICO; UK) or Data Protection Commission (DPC; Ireland) actively regulate to ensure data compliance within the National Health Service (NHS) or Health Service Executive (HSE; Ireland). This article explores the contemporary clinical context and regulatory ramifications within which practicing clinicians are operating. It highlights the need for appropriate guidelines, tools, and education in digital health to enable clinicians to practice in an environment where sensitive patient data can be processed and communicated securely with clarity for patients as to how their data might be used.

## Clinical Communications, Smartphone Technology and APPS

Effective communication is an essential element of good clinical care. However, the traditional methods of communications (e.g., landline telephones, pagers and fax machines) are restrictive. Smartphone technology and communications Apps (including IM) have become ubiquitous in society facilitating cost effective and efficient communication on a “one-to-one” or “one-to-many” basis. For clinicians, the functionality facilitates conversations and consultations between colleagues and enables the sharing of (potentially sensitive) supplementary voice recordings, images and video at the “*touch of a button”* with WhatsApp being the most cited IM application in healthcare (3).

Presumably as a consequence of the magnitude of the COVID-19 pandemic combined with inadequate investment in communications infrastructure, clinicians have been encouraged to draw on their smartphones or Bring Your Own Devices (BYODs) and Apps (5) (e.g., Skype, iMessage, Zoom) for telemedicine activities. Such functionality has positively enabled online/remote consultations, reviews and test result discussions, patient monitoring and clinical photography, including the use of diagnosis Apps from smartphone images. The patient journey and the working life of clinicians have improved whilst pragmatically bypassing the need for time-consuming additional training or purchase of costly new technology at a time of crisis. Regrettably, the perceived advantage of familiarity of these technologies, contrasts with usage of BYODs potentially introducing data security risks for clinicians which they may not be aware of due to a lack of education and training. Of course, not all patients have an awareness of such technologies, or own a compatible device generating inequality in availability to digital services.

## Digital Professionalism

Digital Professionalism may be defined as “*the competence or values expected of a professional when engaged in social, digital communication and mobile technology”* [amended from Oxford English dictionary definition of professionalism (6)].

There are clear challenges for how health professionals conduct themselves in a rapidly evolving digital landscape. It has been suggested that Digital Professionalism education and learning should begin in undergraduate training, “given its relevance at all levels” (7).

This issue has recently been acknowledged in the latest Curriculum for Graduating European Dentists (8) which has an Intended Learning Outcome:

“*graduating dentists must demonstrate digital professionalism by protecting patient data and the appropriate use of social media and digital communication, mindful of how these activities may force them into ethically challenging situations and/or damage the reputation of the wider profession.”*

This healthcare exemplar challenges education providers to best equip its graduates for the challenges of modern digital society. Our anecdotal experience indicates that clinicians have a broad range of attitude and behavior with risky behavior by busy clinicians on the frontline being a concern.

## Record Keeping and Clinical Images

Record keeping of clinical images and “chat messages” stored on BYOD smartphones shared *via* IM and Apps pose a range of serious safety concerns (9). The clinician initiating a dialogue has responsibilities to ensure that any messages/data and responses are recorded in the patient's notes; IM conversations may be subject to Freedom of Information requests or Subject Access Requests (10). However, downloading of the dialogue and any clinical images to store it in the patient notes or digital healthcare record may not be technically possible and even if so, are these data being promptly deleted from the smartphones by all members of the App group? Clinicians may not be aware that smartphones store deleted images in the “*deleted images”* album for 28 days (2). Further, smartphones must be password protected with image streaming to insecure cloud servers and between networked devices turned “OFF” (2, 10). The storage of clinical images using insecure cloud servers associated with a BYOD is concerning (3, 10). Digital images contain additional Exchangeable Image File Format (EXIF) data from geographical co-ordinates to date, time and device details, so anonymising digital clinical images requires a level of digital literacy that many clinicians do not possess or are unaware of.

## Data Security

The “*minimalisation”* of sensitive patient identifiable data is essential when engaged in clinical communications *via* smartphone Apps due to concerns regarding data security (10). Conversely, clear identification of the patient being discussed is necessary for patient safety as communication failure could result in poor clinical outcomes and avoidable errors (11). The conflict between communication accuracy and patient data security arose pre-COVID-19 with patient care prioritized over the security of patient data (12). The potential for a security breach of patient data is heightened when IM Apps use unregulated servers outside the European economic area. Lack of compliance with data protection requirements regarding data mining, fair processing, records management, and the technical challenges posed by BYOD, are well documented (3, 10).

## Cybercrime

Healthcare is one of the most targeted sectors for cybercrime globally due to its rich data source and perception as a “*soft target”* (12). Data vulnerability in healthcare is attributable to limited budgetary resources, fragmented governance, cultural behaviors and through the use of obsolete systems unable to support the latest software/updates (13). Regrettably, high risk and critical vulnerabilities in healthcare systems are increasingly targeted by ransomware and the COVID-19 pandemic anecdotally resulted in an increase in malicious cybercrime activity. An example was the successful access and exploitation of personal and health data from a major ransomware attack on the HSE on 14th May 2021 (14). This was a highly significant attack and the largest known against a health service computer system, with massive negative impact on healthcare delivery. Reports that more was paid to ransomware criminals during the first half of 2021 than for the whole of 2020, statistics that could serve as inspiration to other criminals (14) intent on exploiting health systems. A post incident review commissioned by the HSE provided key learning points for healthcare professions in relation to regular cybersecurity awareness and training for all staff grades (14).

## Data as a Commodity

The business model for many smartphone and App based communications channels involves actively commodifying user data from the device, any networked devices (e.g., fitness trackers) and associated cloud storage. The consequences of “*data harvesting”* by companies such as Meta (owners of WhatsApp and Instagram) and Alphabet (owners of Google, Fitbit, YouTube, Nest) and the “*profiling”* of individuals by exploiting personal data by companies (e.g., Cambridge Analytica) cannot be underestimated or ignored (15). Smartphones have the potential to threaten the security of sensitive patient data and yet, “*the opaque nature of online (including mobile App) tracking, along with the use of data held by mobile operators, is beyond the awareness and understanding of many individuals*” (16). This is acknowledged widely outside of healthcare settings (e.g., auto-manufacturing) where employees are forbidden from using WhatsApp, Snapchat etc. on company issued devices due to data breach concerns (17). Surveillance by Smartphone Apps that harvest the sensitive data, is a genuine concern spanning national, EU and international Data Protection, Information Privacy and Human Rights Laws. Clinicians may be inadvertently enabling the long-term exploitation of patients and their data by third parties (e.g., health insurance companies) or through malicious spyware (e.g., Pegasus).

## Information Governance

UK Guidance to clinicians has evolved significantly over recent years. Advice from the National Health Service (NHSX; with a remit for technology, digital and data sharing and transparency and now part of the NHS Transformation Directorate) in October 2020 (5) stated that “*in the current circumstances it could be more harmful not to share health and care information than to share it*”. Further, they report that “*The Information Commissioner has assured NHSX that she cannot envisage a situation where she would take action against a health and care professional clearly trying to deliver care*”. This assertion is limited to the regulatory action that could be taken by the ICO itself and related specifically to the context of COVID-19. Worryingly, the advice also explicitly endorses the use of “*commercial, off-the-shelf applications such as WhatsApp and Telegram where there is no practical alternative, and the benefits outweigh the risk*”.

NHS Clinicians are currently advised to adopt personal BYOD's for video conferencing consultations, home working and mobile messaging (5). Reassurances have been provided for off-the-shelf tools (Skype/WhatsApp) to be used, with implied consent obtained by patient acceptance of the invitation and entering into the consultation (5). However, intentional in inadvertent sharing of patient data could be unlawful as patients only consent to the video consultation and not for their (sensitive) data to be shared further. A clinician (data controller), cannot simply contract their way out of their obligations to the patient (data subject) under the General Data Protection Regulation (GDPR) (18). Helpfully, the European Commission has provided guidance to EU Member States and App developers on how to comply with GDPR and the ePrivacy Directive (19).

## GDPR and Human Rights

The implementation of GDPR in May 2018, created a single legal framework across EU member states on “*the protection of natural persons with regard to the processing of personal data and on the free movement of such data*” (18). This increased the safeguarding of privacy obligations on organizations that process personal data to undertake significant technical and organizational measures to demonstrate compliance.

The protection of personal information is arguably the most pressing concern in defending fundamental freedoms and human rights (20). If a patient feels that their privacy rights have been violated by a clinician using an inappropriate technology, the ICO or DPC could find their assertion that clinicians are free to use such channels challenged. It is noteworthy that since the UK has left the EU, their data protection regulations are likely to diverge so clinicians trained outside the UK will need training supports to raise awareness of any differences.

It is also important to note that not all patients have equal benefit from or access to these technologies and the relationship with discrimination for underserved and marginalized groups, and health inequality must be acknowledged and explored.

## Conclusion

Smartphone technologies afford clinicians (and their patients) a broad range of highly positive advantages observed acutely during COVID-19. Clinicians have always had the best interests of their patients as a key driver however, the legacy of the well-intended measures undertaken during COVID-19 in relation to patient data and privacy could be felt long after the current emergency ends. Concerns highlighted in this article should be addressed by unambiguous healthcare sector and institutional guidelines with supportive training and education opportunities. Clinicians should be able to access training to optimize appropriate smartphone technologies within regulatory frameworks to overcome data security concerns. Embedding this training into undergraduate curricula, professional CPD requirements and mandatory institutional clinical training would positively encourage digital professionalism.

## Author Contributions

All authors listed have made a substantial, direct, and intellectual contribution to the work and approved it for publication.

## Conflict of Interest

The authors declare that the research was conducted in the absence of any commercial or financial relationships that could be construed as a potential conflict of interest.

## Publisher's Note

All claims expressed in this article are solely those of the authors and do not necessarily represent those of their affiliated organizations, or those of the publisher, the editors and the reviewers. Any product that may be evaluated in this article, or claim that may be made by its manufacturer, is not guaranteed or endorsed by the publisher.
